# Applying Customer Discovery Method to a Chronic Disease Self-Management Mobile App: Qualitative Study

**DOI:** 10.2196/50334

**Published:** 2023-11-13

**Authors:** Nallely Mora, Zoe Arvanitakis, Merly Thomas, Holly Kramer, Elaine H Morrato, Talar W Markossian

**Affiliations:** 1 Department of Public Health Sciences, Parkinson School of Health Sciences and Public Health Loyola University of Chicago Maywood, IL United States; 2 Rush Medical College Rush University Medical Center Chicago, IL United States; 3 Center for Health Innovation and Entrepreneurship, Parkinson School of Health Sciences and Public Health Loyola University of Chicago Maywood, IL United States; 4 Department of Medicine Loyola University of Chicago Maywood, IL United States

**Keywords:** mobile app, disease management, customer discovery, customer segment, value proposition, chronic disease management, self-management, chronic disease, digital health, ehealth, mobile application, mhealth, customer delivery

## Abstract

**Background:**

A significant health challenge is evident in the United States, with 6 in 10 adults having a chronic disease and 4 in 10 adults having 2 or more. Chronic disease self-management aims to prevent or delay disease progression and disability and reduce mortality risk. The evidence to support the use of information technology tools, including mobile apps, web-based portals, and web-based educational interventions, that support disease self-management and improve clinical outcomes is growing. Customer discovery and value proposition design methodology is a form of stakeholder engagement and is based on marketing and lean start-up business methods. As applied in health care, customer discovery and value proposition methodology can be used to understand the clinical problem and articulate the product’s hypothesized unique value proposition relative to alternative options that are available to end users.

**Objective:**

This study aims to describe the experience and findings of academic researchers applying the customer discovery and value proposition methodology to identify stakeholders, needs, adaptability, and sustainability of a chronic disease self-management mobile app (CDapp). The motivation of the work is to make mobile health app interventions accessible and acceptable for all segments of patients’ chronic diseases.

**Methods:**

Data were obtained through key informant interviews and analyzed using rapid qualitative analysis techniques. The value proposition framework was used to build the interview guide. The aim was to identify the needs, challenges (pains), and potential benefits (gains) of the CDapp for our stakeholders.

**Results:**

Our results showed that the primary consumers (end users) of a CDapp were the patients. The app adopters (decision makers) can be medical center leaders including population health department managers or insurance providers, while the consumer adoption influencers (influencers or saboteurs) are clinicians and patient caregivers. We developed an ecosystem map to visualize the clinical practice workflow and how an app for chronic disease management might integrate within an academic health care center or system. A value proposition for the identified customer segments was generated. Each stakeholder segment was working within a different framework to improve patient self-management. Patients needed help to adhere to self-care activities and they needed tailored health education. Health care leaders aim to improve the quality of care while reducing costs and workload. Clinicians wanted to improve patient education and care while reducing the time burden. Our results also showed that within academic medical centers, there were variations regarding patients’ self-reported abilities to manage their diseases.

**Conclusions:**

Customer discovery is a useful form of stakeholder engagement when designing studies that seek to implement, adapt, and sustain an intervention. The customer discovery and value proposition methodology can be used as an alternative or complementary approach to formative research to generate valuable information in a brief period.

## Introduction

Over half of all US adults live with a chronic medical condition such as hypertension, diabetes, dementia, or chronic kidney disease, and almost one-third (27.2%) live with multiple such conditions [1]. Furthermore, these numbers increase with increasing age and pose a serious challenge to health care systems globally with aging populations. Heart disease, cancer, chronic lung disease, stroke, Alzheimer disease, diabetes, and chronic kidney disease are the 7 leading causes of death and disability and are drivers of the nation’s US $4.1 trillion annual health care costs [2].

Chronic disease (CD) self-management aims to prevent or delay disease progression and disability and reduces mortality risk but requires a high level of patient involvement in decision-making and care implementation [3], as well as high levels of health care team support. A core component for self-management of long-term chronic conditions is self-care [4]. Self-care involves actions that individuals take to maintain a healthy lifestyle, including social, emotional, and psychological health, and to prevent further illness or accidents [5]. Patients with chronic conditions are required to accomplish multiple self-care activities each day to maintain health. These daily activities may include taking multiple pills at different times, measuring blood pressure and weight, following a modified diet, and engaging in physical activity, among many other factors. Patients also need to maintain effective communication with their providers. Self-monitoring is one aspect of self-care, and traditionally, primary care providers have asked their patients to track their health data (blood pressure or blood glucose) on paper that they can bring to their next appointment. More recently, technology-assisted disease-monitoring approaches such as health apps can be a safe and feasible alternative [6]. Supporting self-care among patients also involves providing patients with information and skills that are relevant, accessible, and tailored to them [7].

The evidence to support the use of information technology tools, including mobile apps, web-based portals, and web-based educational interventions, that support disease self-management and improve clinical outcomes is growing [8-14]. In 2021, the majority of Americans (85%) owned a smartphone, including 61% of adults aged 65 years and older and 76% of persons with annual household incomes below US $30,000 [15]. About 70% of Americans reported keeping track of at least 1 health indicator, such as weight, diet, exercise routine, or symptoms, through their smartphone [16].

Despite these advances, patients with chronic conditions who would benefit from tracking health indicators with a mobile app do not use such apps on a regular basis [17]. Health app use also varies by patient characteristics and socioeconomic status (SES) [18,19]. Health app adopters and users tend to be younger, White, and from a higher SES group [18]. With an ultimate goal of improving population health equity for chronic disease management, we aim to first understand the stakeholders of a mobile health app for CD self-management and determine its perceived value to them, as described in this paper.

Customer discovery and value proposition design methodology is a form of stakeholder engagement and is based on marketing and lean start-up business methods [20]. As applied in health care, customer discovery and value proposition methodology can be used to understand the clinical problem and articulate the product’s hypothesized unique value proposition relative to alternative options that are available to end users. End users can be patients, clinicians, health care managers, caregivers, health care payors, and others.

Customer discovery methodology has been applied by academic entrepreneurs and researchers to stimulate and sustain health innovations, mostly in the health informatics domain [21-23]. Using customer discovery as an essential tool for understanding the needs of stakeholders and a product’s value proposition is core to the Innovation-Corps at the National Center for Advancing Translational Science (I-Corps@NCATS) entrepreneurial training program. I-Corps@NCATS is funded by the US federal government and is modeled on the original National Science Foundation Innovation-Corps (I-Corps) training program from 2011 [24]. The curriculum trains health researchers on how to articulate the impact of their innovation. The product’s value proposition states the differentiating benefits customers can expect versus what is currently available from competing alternatives, including the usual care (standard of care). The value proposition serves as a business hypothesis to be validated through stakeholder engagement and customer feedback. This is referred to as product-market-fit, which is when there is evidence that the product or service actually delivers the hypothesized value in the market and there is growing customer demand [25].

Some propose that major changes in the lives of customers can be achieved through three processes [26]: (1) determining what the customer wants to accomplish (what is the job to be done?), (2) defining what success is for the customer at every stage along the transformation journey, and (3) identifying and addressing the barriers to transformation. To develop products or services which truly promote health, for example, mobile apps for common chronic diseases (eg, diabetes, hypertension, and others), we needed to learn from past lessons and apply key principles of customer discovery, value proposition design, and transformation. Modern tools, and in particular digital and other technology tools, can be leveraged to effectively engage customers through remote web-based community development [27]. Yet, such digital tools may not be sufficient for the customer experience to be fully realized or optimized [28], and novel and broad approaches to engage customers are needed. Stakeholder engagement is a building block of interventions that can enhance adoption, reach, implementation, and sustainability [21,29,30].

This paper describes the experience and findings of academic researchers from applying the customer discovery and value proposition methodology within the I-Corps@NCATS program for developing a tailored mobile app for CD management, which we called a chronic disease self-management mobile app (CDapp). The overall objective is to describe our experiences and findings in identifying the roles and value propositions for different stakeholder segments. The motivation of our work is to improve health app acceptability and adoption among all segments of patients with CD, with a focus on improving health equity, given that many of the major CDs affect disadvantaged populations.

We hypothesize that the CDapp can successfully support disease self-management among patients with multiple CDs and that each stakeholder segment will have unique roles and barriers when adopting the new technology. From this hypothesis, an initial value proposition statement is generated separately for patients and clinicians.

In the case of patients, CDapp with usual care case management can help patients with multiple chronic conditions to self-manage their disease by improving adherence to self-care activities and health education. This is because self-tracking of health data and adhering to self-care activities without the support of CDapp can be overwhelming to patients.

Whereas in the case of clinicians, CDapp will support clinical teams in educating patients and increasing access to patient’s health data. Disease management and health education to all patients in a practice are resource intensive, and CDapp can be a tool to facilitate disease management and health education.

## Methods

### Overview

The study design was based on the customer discovery and value proposition methodology [24] and aimed to identify stakeholders’ perceptions of the most important “jobs to be done” to help individuals with CD self-management. The stakeholders included individuals with multiple CDs, their caregivers, and health care team members. We aimed to identify critical “pains” and “gains” for self-management (challenges and potential benefits) and how to measure success as defined by the stakeholders. We aimed to learn the wants, pains, and gains of individuals living with chronic conditions from all sociodemographic and economic backgrounds and those who support the health of these patients, including health care providers, health system administrators, information technology personnel, and caregivers.

The qualitative approach was used for data collection and analysis. We defined participants broadly, including individuals with CDs defined as having 2 or more chronic conditions that lasted more than 6 months, as well as their caregivers, primary care providers, population health department managers, hospital health information administrators, and pharmaceutical industry leaders. Interviewees were recruited through a combination of purposive and snowball sampling. Specifically, participant identification and interviews were conducted by 3 investigators on the team (authors NM, ZA, and TWM). These investigators are housed at 2 different academic medical centers in the Chicagoland area, which is a highly competitive market in the health care industry. Recruitment efforts were limited to the social and professional networks of these investigators. As such, potential participants were drawn from the personal professional networks of the investigators and previous research participants who were enrolled in other institutional review board–approved studies at our institution and consented to be contacted. The provider and other contacts included primary care providers who have collaborated previously with the research team and other management-type contacts from our existing professional networks.

Using the customer discovery framework, we developed an interview guide asking about current practices, definitions of success, perceived barriers, opportunities for improvement, and facilitators (what works well), in relation to CD management and relative to the specific role of the stakeholder in the health care ecosystem. Due to the I-Corps@NCATS program structure, which is within a training program, and a limited data collection period of 4-5 weeks, we limited our ecosystem to academic medical centers [31]. Academic medical centers deliver a large portion of care for older adults who carry the majority of the burden of CDs, including beneficiaries within Medicare’s accountable care organizations. These centers are also known to serve as safety net health systems for patients with lower SES, and 2 academic institutions of the interviewers have been nationally recognized for their work with such patient populations.

All questions were open-ended to elicit detailed responses and to allow for the discovery of novel ideas that may not have been considered by the interviewers (Multimedia Appendix 1). We pilot-tested the interview guide among 3 participants and adjusted it as needed in an iterative process. Interviews were conducted on a one-to-one basis by 1 of the 3 team members (NM, ZA, and TWM) either by phone or by Zoom (Zoom Video Communications, Inc). Phone interviews were recorded when possible and transcribed immediately after the interview. Zoom interviews were transcribed by Zoom app and edited after the interview.

### Ethical Considerations

The study met the definition of quality improvement and was approved by Loyola University Chicago’s institutional review board (IRB number 215918060322). For participants who elected to participate in the interviews, we read a research information sheet that described the nature of the project and the voluntary nature of participating in the brief interview. Interviewees verbally agreed to participate at the start of the interview and verbal consent was documented. There was no compensation for participating in the study.

### Data Collection

Data collection took place between October and November 2021. Based on the I-Corps@NCATS program recommendation for the number of interviews, we targeted a total of 30 interviews. Due to limited time, scheduling constraints, and the ongoing pandemic, this number was not reached. All our clinician and health care leader interviews were conducted with individuals who worked at academic medical centers. Transcripts from all interviews and interviewer notes served as the primary data source for the analysis. No identifiers were requested, and the data were maintained on a secure server that is accessible only to the primary investigators (NM and TWM), for future reference.

### Data Analysis

Interview records were analyzed by 2 investigators (NM and TWM) using a rapid, inductive qualitative approach, which included the following steps: (1) creating a matrix for codes, (2) establishing interrater reliability by independently coding 3 interviews and generating consensus, (3) independently coding the remaining interviews, and (4) summarizing themes [32-34]. Next, data were grouped by the type of interviewee to help identify relevant themes within the customer discovery and value proposition framework and guide the initial coding system. Coding analysis was supported by NVivo 12 qualitative data analysis software (QSR International Pty Ltd, 2020). Team members (NM, ZA, and TWM) met regularly (synchronously and asynchronously) during the interviewing and coding process to review, interpret the coded transcripts, and identify the customer segments and their value propositions. Our coding and learning process was iterative, toggling between stakeholder and customer segments and deriving the unique value proposition for each stakeholder and customer segment. I-Corps faculty and content experts meet regularly with team members to review progress and provide feedback.

## Results

### Overview

We conducted a total of 23 interviews. Our interviews included population health department managers (n=3), system information technology specialists (n=2), persons with leadership positions in health care (n=2), clinical care providers (n=6), and patients with CDs (n=10).

Our results showed that the primary consumers (app users) of CDapp were the patients. App adopters (decision makers) can be medical center leaders including population health department managers, in addition to insurance providers. Consumer adoption influencers (influencers or saboteurs) were clinicians and patient caregivers (Tables 1 and 2). Our interviewees highlighted that in order to have an app adopted, it needed to show multipronged value specific to each customer segment.

In the following sections, we described the major themes we identified from the interviews and organized by stakeholder segment.

**Table 1:** Summary of customer discovery for chronic disease self-management mobile app learning results by stakeholder segment (N=23).

Customer discovery	Targeted stakeholder							
	Consumer (app user)	Adoption influencer				Customer (app adopter and decision maker)		
	Older patients with chronic conditions (2 or more)	Clinical care providers (primary care providers, specialists, registered nurses, and nurse practitioners)		Caregivers^a^		Medical center leadership (population health department managers, and information technology specialists, and case management administrators)		Insurance providers^a^
Jobs to be done	Adhere to self-care activities. Self-care activities may include taking multiple pills, measuring blood pressure and weight, following a restricted diet, and engaging in physical activity.	Ensure patients understand their diagnosis, medication schedule, and necessary lifestyle changes. Review health data (eg, blood pressure and blood glucose) recorded or summarized by patients during the clinic encounter. Screen for possible chronic conditions (eg, cognitive decline)	Help older patients with self-care activities.		Manage the health of patients within the Accountable Care Organization contracts. Reduce readmission rates within the CMS’^b^ HRRP^c^. Provide case management for patients with a high risk of poor health outcomes.		Cover health care costs of beneficiaries and earn profits.	
Pains to be solved	Some patients have a chaotic life or have limited primary resources (eg, food), making disease management not a priority. A large majority of patients lack health literacy.	Manage multiple conditions during brief clinic visits. No time for patient education about lifestyle modification during a clinic visit. Health data that patients bring to the office visit can be overwhelming and complex. Lack of access to pharmacist expertise for drug reconciliation, whether in person or using software.		During the COVID-19 pandemic, many family caregivers needed to provide heightened levels of homecare management.		High readmission rates within the HRRP. Fragmented care management that is based on each condition separately.		Patients with chronic conditions have high health care costs.
Emotional gains to be achieved	Improve self-confidence in managing their condition. The good feeling that they are doing everything they can to improve their health.	Seeing progress in patients’ health (eg, controlled blood glucose and controlled blood pressure) is gratifying.		Peace-of-mind that their loved one’s care needs are being well managed.		Reputation of the medical center (affecting referrals).		Maintain a reputation as an ethical insurance company.
Barriers and facilitators	Barriers and facilitators: there is a large variation regarding patients’ abilities to manage their disease, some do very well, and some do not. Facilitators: Some patients identify health apps in Google and Apple iOS stores to help manage self-care activities. Facilitators: patients do want to learn more on how to better manage their condition. Facilitators: not all patients are satisfied with their self-management approach.	Barriers: providers have accepted that there is not much they could do regarding promoting lifestyle modification for their patients. Facilitators: open to prescribing apps with scientifically proven effectiveness.		Facilitators: available support groups.		Barriers: care management services are most times paid out-of-pocket, and patients do not follow through because of the out-of-pocket costs. Facilitators: care coordination or management services are available for selected populations, for example, Medicare, Medicaid, or the HRRP program. Facilitators: availability of certified diabetic educators for selected populations, for example, Medicare patients. Facilitators: promoting health app use can be incorporated into care management.		Not all health insurance companies cover care management, but some do for specific illnesses, such as diabetes.

^a^Reflecting perceptions that were expressed by our interviewees regarding other roles.

^b^CMS: Centers for Medicare and Medicaid Services

^c^HRRP: Hospital Readmissions Reduction Program.

**Table 2:** Illustrative quotes. Summary of customer discovery learning results by stakeholder segment.

Targeted stakeholder			Illustrative quotes
**Consumer (app user)**			
	Older patients with chronic conditions (2 or more)	“Patients will individually identify apps.” [PCP] “I am not necessarily satisfied (with my self-management)” [Patient]	
**Adoption influencer**			
	Clinical care providers (PCPs^a^, specialists, registered nurses, nurse practitioners)	“I haven't given a whole lot of thought to that (disease management) because it's just you adapt to working within the system, you're in.” [PCP] “In order to reach the level that you could prescribe... need study that shows app is helpful.” [PCP] “I write prescriptions for the continuous glucose monitors that are connected to apps where you can write your diet...” [PCP]	
	Caregivers^b^	—^c^	
**Customer (app adopter and decision maker)**			
	Medical center leadership (population health department managers, information technology specialists, and case management administrators)	“I do not engage with those tools...but that could be incorporated into care coordination model...” [PCP]	
	Insurance providers^b^	“You refer someone with diabetes to a diabetes educator, sometimes insurance company has something they add or not, for most other chronic diseases, they do not.” [PCP]	

^a^PCP: Primary care provider.

^b^Reflecting perceptions that were expressed by our interviewees regarding other roles.

^c^Characteristics or phenomena that were not observed in the interviews.

### Theme 1: Limited Adaptation of Disease Management Tools

Interviewees noted that although academic medical centers and other community health centers are using various tools, but the practice is not widespread.

Only about 25% of patients follow through with those [care management] referrals or I don't refer because of the cost.

Primary care provider (PCP)

I do not engage with those tools...but that could be incorporated into care coordination model...

PCP

We talk about an approach, lifestyle and medicines that are on the table.

PCP

### Theme 2: Barriers to Adaptation of Tools

We heard from clinicians that patients with lower educational attainment and income were the ones who struggled the most with disease self-management. However, along with low health literacy, clinicians believed that these patients were not ready to adopt a new technology, which remains a pressing problem to be solved.

In our practice, we have some people who have really chaotic lives, and are very challenged, while others whose things are organized and really you know they're on top of it.

PCP

Many of them are in their 80s, they’re older patients who you know, some of them don't even have a college education so they don't have the resources to go on to the Internet and look and see how they can help themselves, or they don't even have access, for example, to a scale, or they don't even have access to healthy food.

PCP

### Theme 3: Willingness to Adopt New Tools by Patients

Patients with chronic diseases and clinical providers indicated that they may consider adopting a novel tool to address chronic disease self-management.

Once they (patients) understand and they know what happened, they can play a very important role in managing their disease...

PCP

If my doctor asked me to use the app, I will certainly give it a try!

Patient

### Theme 4: Interest in Patient Education via Apps

Clinicians expressed interest in having tools for patient education in an app. The time per encounter is brief necessitating attention to only the most pressing clinical issues. However, they were apprehensive of assuming responsibility and of information overload from health data generated by the app.

[I have a] love and hate relationship with health data.

PCP

My part is to tell the patient what they can do...once they know, it’s their job...to go home and manage...

PCP

### Theme 5: Systems Perspective for Disease Management

Health care leaders recognize that the PCPs have limited time per encounter and need support from their organizations’ leadership to be successful with CD management, especially for those patients who are at high risk of poor health outcomes. Case managers follow up with high-risk patients to help educate and support disease self-management. Some insurance companies have case managers for patients identified as high-risk and their goal is to decrease health care costs of their beneficiaries.

Definitely need support somehow that you're not going to get from a 15-minute doctor's visit to support them to manage their care to prevent re-hospitalization, there is a gap after hospitalization.

Medical center leadership

Not the single chronic disease, which is rare, the multiple chronic conditions pose the real challenge, two-third of 65+ years old group have chronic condition(s), of which half have 5+ conditions.

Medical center leadership

### Theme 6: Compliance With Governing Requirements

Health care professionals shared that achieving the triple aim benchmarks set by governing agencies is an important motivating need. These aims are to improve quality, outcomes, and reduce per capital cost, especially for patients at higher risk for readmission [35].

We [PCPs] are being monitored as well as the health system is being monitored.

PCP

[We are] really more interested because we see that helping people manage their chronic diseases, how that helps them downstream from avoiding a lot of complications and hospitalization.

Medical center leadership

**Figure 1:**
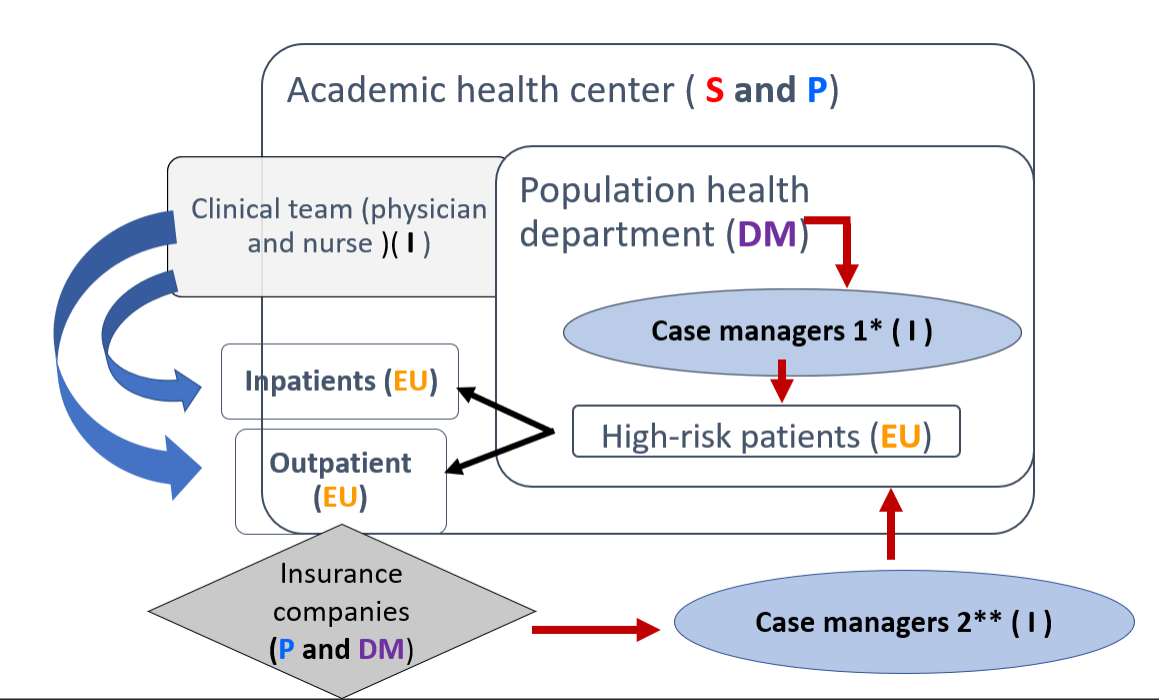
Healthcare ecosystem process map for Chronic Disease Self-Management Mobile Application.

Tables 1 and 2 summarize the customer discovery results by stakeholder segment and additional exemplar quotes. Table 3 presents the updated value proposition statements by customer segment based on our learning. An ecosystem map was developed to visualize the clinical practice workflow and how an app for CD management might integrate within an academic health care center or system (Figure 1). The ecosystem map focuses on the value proposition use case scenario in which the primary target consumers (end users) are high-risk older patients either discharged from an inpatient setting or seen in an outpatient setting. These patients are usually managed by case managers, who are assigned either by the health system or health insurance companies. Health system leadership and insurance companies are therefore the app adopters since they are decision makers on whether to adopt the CDapp in their practice. The case managers and clinicians are influencers for the adoption and use of CDapp because they could recommend CDapp in practice or champion CDapp to their leadership.

Our initial hypotheses were mostly supported and supplemented with more specific value propositions per customer segment.

**Table 3:** Target value proposition for a chronic disease self-management mobile app (CDapp) by customer segment.

Customer segment and stakeholder role			Value proposition
**End user**			
	Patient (app user)	Providing tailored health information. Unlike existing free health apps, the CDapp will track health data and give access to empirically supported disease promotion tools and education that are accessible to patients.	
**Adoption influencer**			
	Clinical care providers	Providing a summary of health data that clinicians can review during the clinical encounter. Unlike unstructured patient self-reported data (based on memory or on a paper sheet), summary health data can be available in CDapp at the time of the encounter. In addition, the CDapp can provide targeted educational tools for patients versus the status quo of “no time” for education during the clinical encounter.	
	Caregivers	Providing alternative support systems for patients to help relieve caregiver burden and uncertainty. In addition, the CDapp provides a means for supporting efficient clinical encounters in which the caregiver accompanies the patient (because everyone can see the same health data).	
**App adopter and decision maker**			
	Medical center leadership	Providing support to existing care management activities to further facilitate meeting the triple aim, which involves improving the patient experience of care, improving population health, and reducing per capita cost. Helping to meet the quality metrics set by national agencies and other payors and reduce penalties for low-quality care by improving patients with multiple chronic with self-management.	
	Insurance providers	Decreasing future health care use and cost by supporting beneficiaries’ disease self-management.	

## Discussion

In this paper, we describe the customer discovery methodology as it applies to adopting a CDapp in clinical practice. Our work is differentiated by taking a customer development model approach to discover what value proposition is necessary for supporting a scalable and sustainable intervention.

Through the customer discovery process, we learned that for a CDapp to be adopted, it must satisfy the needs of multiple stakeholder segments: target consumers (end users) who are older patients with multiple complex comorbidities; target customers (purchasing decision makers) who are medical center leaders; and adoption influencers (advocates or saboteurs). Ecosystem mapping visualizes how the different stakeholders involved affect the adoption of a new digital health intervention related to one another (Figure 1). The main decision makers who would pay for the intervention included population health department managers of medical centers, whose costs could potentially be underwritten by health insurance companies. Providers were influencers who could champion the app to administration and prescribe the app during clinical practice. These providers were not enthusiastic about health apps unless they included health education and eased, rather than increased, their workload. Although we did not target caregivers for our interviews, some patient interviewees were accompanied by their caregivers and the concept of caregivers emerged on multiple occasions during our interviews. These caregivers support patients’ needs in adopting or using health technologies and they can either be end users or influencers. Effective health technology interventions may alleviate caregiver burden by providing alternative support systems to patients.

Importantly, we identified that our stakeholders, within the context of our academic medical centers, had common needs—namely, to improve patient self-management and quality of care. However, these stakeholders also had different minimum requirements. Patients needed easy-to-use and understand tools to help them feel more confident about managing their conditions; clinicians needed an efficient means to improve their individual performance on quality assessment metrics while minimizing any incremental time burden; health system leaders needed to improve institutional performance on quality-of-care metrics while reducing costs and minimizing incremental workforce effort. Clinical providers stated that patient education is necessary for improving patient self-care and that is important information because these tools can be incorporated in CDapp.

We identify key barriers and facilitators to inform future adaptations of the technology and to identify target segments and key strategies for the successful implementation of the mobile app into care pathways of CD management. Our discovery process guided us to focus on two main consumer-customer archetypes: (1) the older patient with multiple CDs and (2) population health department managers (Textboxes 1 and 2).

**Customer archetypes for a chronic disease self-management health app to improve health equity (adopters).**

**Older patient with multiple (2 or more) chronic conditions**

- Mr. X is a patient with hypertension and diabetes. He is 68 years old. He is usually good at managing his blood pressure and diabetes, but he struggles to keep motivated to exercise, eat healthy, and follow his doctors’ recommendations. He also thinks the doctor’s visit is too brief and he is not sure he took notes of all the details during the visit, and he wishes he could learn more about managing his health. If Mr. X does not manage his health properly, there is a high likelihood that he may get Chronic Kidney Disease. His wife Mrs. X helps him at home with disease self-management. They have searched the Apple and Google stores and the internet to find various tools to help with self-management. He has an app for diabetes on his phone, another for weight, and a third for physical fitness. He also searches the internet for health tips and support groups.
- Ms. Y is a patient with hypertension and chronic kidney disease. She is 70 years old. She has an Android phone, but she rarely uses her phone other than making calls. Ms. Y lives alone, and she struggles to pay her bills, which raises a concern about whether she is starting to experience memory problems and the early stages of dementia. She has told her provider that she missed taking her medications to cut costs. She has one daughter who lives out of state and one son who visits her sometimes. Ms. Y has missed health care appointments because she does not have reliable transportation. She likes having walks, but her neighborhood is not safe for walks, and she doesn’t have an accessible community or recreation center in her neighborhood. The near future is looking grim for Ms. Y because if she does not take care of her health, she is likely to experience a cardiovascular event or get on dialysis.

**Customer archetypes for a chronic disease self-management health app to improve health equity (decision maker: payor).**

**Academic medical centers**

- These centers are serving disproportionately higher percentages of lower-income patients and patients from multiple racial and ethnic backgrounds. They may be located in lower-income neighborhoods. A large percentage of their patients may be impacted by disparate social determinants of health. These centers are struggling to meet National Committee for Quality Assurance (NCQA) metrics for primary care patients (medical home); Centers for Medicare and Medicaid Services’ hospital readmissions reduction program for hospitalized patients (readmissions metrics); and Medicare, Medicaid, and selected insurance companies Accountable Care Organizations (ACO) that involve maintaining quality and saving costs. They use case managers for various programs, who would use health apps for patient disease management.

Advancing health equity is an essential component of health care quality [36], and CDapp can also be 1 of many tools available to health care leaders to advance health equity. We anticipated patients with lower educational attainment and income were the ones who struggled the most with disease self-management [37]. As part of our customer discovery process, we aimed to comprehend the needs, pains, and gains of individuals with chronic conditions who are usually older and not necessarily technology or health-savvy. This aspect may distinguish our work from the customer pool of other apps in the Apple and Google stores that typically target younger individuals who are information technology and health literate and are often eager to adopt new tools even if complex to use.

Customer discovery is a form of stakeholder engagement. Using customer discovery as a methodology in the research design of studies that seek to implement, adapt, and sustain an intervention could provide valuable information that can be achieved in less time than conventional qualitative research methods. Furthermore, the flexible framework for data collection allowed us to use data from all interview participants (for example, caregivers accompanying patients), which would often not be possible in strict research study protocols. We found the less formal and expedited approach of scheduling and completing interviews within the customer discovery and value proposition methodology novel to generate valuable information in a brief period of time.

Our study has limitations. Interviewers had no previous experience in using customer discovery as a tool despite their expertise in research methods including qualitative methods; however, the interviews were conducted in the context of the I-Corps@NCATS training program, and the interviewers were coached by the instructors throughout the process. The training program used a sprint format which posed challenges to schedule interviews within a few days, especially with hospital administration and clinicians. Future steps for our group will be to conduct more interviews focusing on the identified customer segments to explore strategies for the sustainability of the health app intervention. Subsequent research will pilot the app and generate supporting evidence of its benefits using a combination of qualitative and quantitative (mixed methods) data collection to further validate the app’s value proposition in real-world settings.

Due to the scope of the project, we limited our ecosystem to academic medical centers, knowing that academic medical centers deliver a large majority of care in urban settings for our target population of older patients with complex conditions. Additional work can be done to explore these concepts within community health centers and less urban locations. Last, additional work needs to be conducted to identify facilitators and barriers from the perspective of health care leaders in integrating a CDapp into the electronic health record, which is a necessary major component for CDapp adoption in clinical practice.

The customer discovery methodology is an intervention development tool for researchers who aim to develop a product or service within a health care setting. This methodology allows to elicit insights from a range of stakeholders, elucidating both the perceived and real needs of stakeholders; defining what success means for stakeholders; and identifying barriers and facilitators to adoption, implementation, and sustainability. In our case, the use of the methodology helped us to narrow our customer segments and understand their needs, clarified what a successful self-management tool would include, and helped identify facilitators and barriers for integrating a CDapp into the clinical workflow according to different stakeholders.

## Appendix

Multimedia Appendix 1 Customer discovery interview guide.

## Abbreviations

CD: chronic disease
CDapp: chronic disease self-management mobile app
I-Corps: Innovation-Corps
I-Corps@NCATS: Innovation-Corps at the National Center for Advancing Translational Science
PCP: Primary care provider
SES: socioeconomic status

## Data Availability

The data generated or analyzed during this study are available from the corresponding author upon reasonable request.
