# Supplementary material for: Applying Customer Discovery Method to a Chronic Disease Self-Management Mobile App: Qualitative Study
Source: JMIR Form Res. 2023 Nov 13;7:e50334. doi: 10.2196/50334 (PMC10682919; doi:10.2196/50334)
Supplement: Multimedia Appendix 1 [file formative_v7i1e50334_app1.docx]

**Customer discovery interview guide**

We want to ask you a few questions about how individuals and providers manage chronic medical conditions like diabetes, heart disease, cognitive dysfunction, and kidney disease.

1. Do you manage chronic conditions? Which ones?
2. How do you manage a/your chronic condition? Please describe the process.
3. Are you satisfied with your current management?
4. Do you think it needs improvement? Why, what needs improvement, and do you have ideas about how?
5. Do you use tools or support systems (mobile app, online support tools, pill box) in managing chronic conditions, what are these, and what do you like or dislike about them?
6. Is there any other information you want to share, in other terms, what did I forget to ask?
7. Can you think of another person who may have information relevant for me?
